# From Penicillin-Streptomycin to Amikacin-Vancomycin: Antibiotic Decontamination of Cardiovascular Homografts in Singapore

**DOI:** 10.1371/journal.pone.0051605

**Published:** 2012-12-12

**Authors:** Wee Ling Heng, Chong Hee Lim, Ban Hock Tan, Maciej Piotr Chlebicki, Winnie Hui Ling Lee, Tracy Seck, Yeong Phang Lim

**Affiliations:** 1 National Cardiovascular Homograft Bank, Department of Cardiothoracic Surgery, National Heart Centre Singapore, Singapore, Singapore; 2 Department of Infectious Diseases, Singapore General Hospital, Singapore, Singapore; 3 Inpatient Pharmacy, Singapore General Hospital, Singapore, Singapore; Beijing Institute of Microbiology and Epidemiology, China

## Abstract

**Background:**

In February 2012, the National Cardiovascular Homograft Bank (NCHB) became the first tissue bank outside of North America to receive accreditation from the American Association of Tissue Banks. From 2008 to 2009, NCHB had been decontaminating its cardiovascular homografts with penicillin and streptomycin. The antibiotic decontamination protocol was changed in January 2010 as amikacin and vancomycin were recommended, in order to cover bacteria isolated from post-recovery and post- antibiotic incubation tissue cultures.

**Aim:**

The objective of this study is to determine the optimal incubation conditions for decontamination of homografts by evaluating the potencies of amikacin and vancomycin in different incubation conditions. Retrospective reviews of microbiological results were also performed for homografts recovered from 2008 to 2012, to compare the effectiveness of penicillin-streptomycin versus the amikacin-vancomycin regimens.

**Methods:**

Based on microbiological assays stated in United States Pharmacopeia 31, potency of amikacin was evaluated by turbidimetric assay using *Staphylococcus aureus*, while vancomycin was by diffusion assay using *Bacillus subtilis* sporulate. Experiments were performed to investigate the potencies of individual antibiotic 6-hours post incubation at 4°C and 37°C and 4°C for 24 hours, after the results suggested that amikacin was more potent at lower temperature.

**Findings:**

Tissue incubation at 4°C for 24 hours is optimal for both antibiotics, especially for amikacin, as its potency falls drastically at 37°C.

**Conclusion:**

The decontamination regimen of amikacin-vancomycin at 4°C for 24 hours is effective. Nevertheless, it is imperative to monitor microbiological trends closely and evaluate the efficacy of current antibiotics regimen against emerging strains of micro-organisms.

## Introduction

Clinical use of aortic and pulmonary valve homografts has been limited primarily by their chronic shortage due to rarity of donors. Moreover, the recovery of micro-organism-free homografts can be challenging, as this is dependent on several factors, such as bacterial proliferation post-mortem, environmental factors at the recovery site and aseptic techniques during homograft recovery. This led to the development of various decontamination methods employed by different heart valve banks.

According to Brockbank et. al., there are four eras in the history of homograft treatment for use in implantation in humans. In the first era, fresh aseptically recovered homografts were used, with implantation taking place within hours or days of recovery. In the second era, there was the extensive experimentation on various decontamination and storage techniques. Harsh methods of decontaminating homografts were explored, such as high concentration antibiotic incubation, gamma irradiation and chemical decontamination using formaldehyde, glutaraldehyde, beta- propriolactone and ethylene oxide. Although these techniques increased the availability of homografts, valve durability was adversely affected, resulting in poor clinical outcomes among patients. This caused a waning in interest in the use of such homografts for implantation. Gradual popularity of antibiotic-treated refrigerated homografts marked the third era, where aseptically recovered homografts were treated with antibiotics and stored in various culture media at 4°C for up to 6 weeks. These milder techniques improved valve durability and ultimately, patient outcome. Finally, the current era uses a combination of techniques, from aseptic homograft recovery to low-dose antibiotic decontamination, followed by cryopreservation and storage of the homografts in liquid nitrogen [1].

To prevent microbial transmission to the recipient, most cardiovascular homograft banks decontaminate the homografts with antibiotics. However, they vary in types, concentrations, incubation durations and temperatures, as currently, there is no consensus on an optimal formula [2], [3]. Differences in practice could probably be attributed to the differences in local microflora as well as individual tissue banks’ experience and preferences. Despite the variations, reported rates of success in decontamination from different banks remain comparable at between 60% to 70%. This translates to a loss of approximately 30% of potential homografts due to decontamination failure. Hence, to meet the rising clinical demand for cardiovascular homografts, more effort is required to improve decontamination efficiency [2].

From 2008 to 2009, NCHB adopted the antibiotic regimen consisting of low concentration penicillin G and streptomycin (50 IU/mL and 50 ug/mL respectively). Homografts were incubated at 37°C for between 6 to 12 hours, in a nutrient medium, Medium 199 (M199), containing antibiotics. This regimen was effective until a homograft was tested positive for MRSA in a post-recovery tissue culture. Although post-antibiotic incubation cultured negative for microbiological growth, it prompted NCHB to review the effectiveness of its current antibiotic regimen against micro-organisms isolated from our homografts, as penicillin and streptomycin are ineffective against MRSA and other resistant strains of bacteria.

Given the rising problem of antibiotic-resistant micro-organisms, Infectious Diseases physicians and pharmacist from the Singapore General Hospital (SGH) recommended the use of amikacin and vancomycin for decontamination against local microflora. The recommended concentrations for decontamination of homografts are at concentrations of 100 ug/mL for amikacin and 50 ug/ml for vancomycin [4]. Before implementing this new regimen, NCHB performed studies to determine the optimal incubation condition for both amikacin and vancomycin. In this report, we describe the results of these studies. We also compare the effectiveness of penicillin-streptomycin and amikacin-vancomycin combinations.

## Materials and Methods

### Reconstitution and Dilution of Antibiotics

To prepare the antibiotic solution for the experiments, the antibiotics were reconstituted and diluted to the concentration for decontamination – 100 ug/ml for amikacin (Lisapharma, Erba Italy) and 50 ug/ml for vancomycin (Cheil Jedang, Seoul Korea).

A vial of 2-ml of 500-mg amikacin was opened. 0.1 ml of amikacin was removed and injected into 250 ml of M199. A vial of 500-mg of vancomycin hydrochloride powder was reconstituted by dissolving it with 10 ml of water for infusion. After complete dissolution of the powder, 0.25 ml of the reconstituted vancomycin solution was transferred to the other 250 ml of M199. The antibiotic solutions were mixed thoroughly. These procedures were performed aseptically.

### Incubation Condition of Antibiotics

The test antibiotics were subjected to the following incubation temperatures and duration to examine the optimal decontamination condition for homografts. Experiments were performed to investigate the individual antibiotic potencies of amikacin and vancomycin after 6-hours incubation at 4°C and 37°C. We also investigated the antibiotic potencies after incubation at 4°C for 24 hours after we had shown that amikacin is more stable at this temperature.

Three sets of antibiotic solutions were prepared. Each set of antibiotic solutions consisted of (1) two freshly prepared tubes of antibiotic solution containing amikacin and (2) two tubes of antibiotic solution containing vancomycin. For the first experiment, the first set of antibiotic solutions was incubated at 4°C for 6 hours in a pharmaceutical refrigerator whereas the second set was incubated at 37°C for 6 hours in a 37°C dry oven. A fresh tube each of amikacin and vancomycin were tested as a control.

### Antibiotic Potency Assays

The potencies of amikacin and vancomycin were evaluated by comparing the inhibition of sensitive micro-organisms caused by known concentrations of the test antibiotics and their standard reference. The microbiological assays were performed in August 2009 according to the United States Pharmacopeia (USP) 31 <81>.

### Amikacin – Turbidimetric Assay

The potency of amikacin was demonstrated using turbidimetric assay. It is based on the inhibition of growth of *Staphylococcus aureus* (ATCC 29737) culture in a liquid medium (peptone 6.0 g, pancreatic digest of casein 4.0 g, yeast extract 3.0 g, beef extract 1.5 g, dextrose 1.0 g). The turbidity of the medium, which is directly proportional to the density of micro-organisms and inversely proportional to the activity of antibiotics, was evaluated by reading at optical density of 530 nm.

The first part of the experiment involved the preparation of standard references in triplicates for 5 different concentrations of amikacin at 40 ug/ml, 35 ug/ml, 30 ug/ml, 20 ug/ml and 10 ug/ml. 1 ml of standard reference solution was added to 9 ml of inoculum with micro-organism culture in each tube. Two control tubes, containing 9 ml of inoculum with 1 ml of water, were prepared. The tubes were placed in a rack to incubate between 36°C to 37.5°C for 17 hours in a dry oven. After incubation, 0.5 ml of 0.9% formaldehyde was added to each tube. In addition, a tube containing 0.5 ml of 0.9% formaldehyde was used as a “blank” to set the spectrophotometer to zero. The absorbance of each tube was read in a spectrophotometer (UV-2450 Shimadzu UV-Vis Spectrophotometer) fitted with a 530-nm filter. A straight-line graph was plotted based on the mean absorbance results obtained from the standard references, with log dose of antibiotics as the x-axis and the absorbance as the y-axis.

The second part involved the evaluation of test amikacin after incubation in various test conditions, in accordance to “Incubation Condition of Antibiotics”. Similar to the preparation of standard references, amikacin were prepared in 5 different concentrations of 40 ug/ml, 35 ug/ml, 30 ug/ml, 20 ug/ml and 10 ug/ml. The tubes were placed in a rack to incubate between 36°C to 37.5°C for 17 hours. The absorbance of each tube was read at optical density of 530 nm. The log doses of amikacin after incubation in various test conditions, were interpolated from the optical density readings obtained, from the standard reference graph. The log dose readings will then be anti-logged (*D_α_* ). The formula of calculating the percentage potency was as follows:
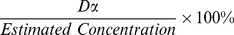



The results obtained were analysed for statistical significance using two-tailed t-test, with the assumption that the population has equal standard deviations (SDs). The results are considered significant if the p-value is less than 0.05.

### Vancomycin – Cylinder Plate Assay

The potency of vancomycin was determined using the cylinder plate diffusion assay. The assay is based on the diffusion of antibiotics solution from the cylindrical disc through a solidified agar layer in a petri dish. The diffusion of antibiotics from a cylindrical disc through a solidified agar medium resulted in the inhibition of growth of *Bacillus subtilis* (ATCC 6633) sporulate. A clear circular zone of microbial inhibition around the disc containing antibiotics solution would form. The diameter of the inhibition zone, which directly correlates to the potency of antibiotics, was subsequently measured.

Agar plates were lawned with 10^5^ to 10^6^ cells/ml *Bacillus subtilis* sporulate suspension for the analysis. The seed layer of inoculum was added on the agar plates. The plates were tilted back and forth to spread the inoculum evenly over the surface. The inoculum was allowed to dry. Six assay wells were created on the inoculated surface on each plate, 3 on each half of the plate with a uniform spacing, of radius 2.8 cm per well. 20 ul standard reference was transferred into each of the 3 wells on the left half of the plate. The other 3 wells on the right half of the plate were then filled with 20 ul of the corresponding concentration of test vancomycin. The test vancomycin were incubated at various test conditions, in accordance to “Incubation Condition of Antibiotics”. Five different vancomycin concentrations of 15.6 ug/ml, 12.5 ug/ml, 10 ug/ml, 8 ug/ml and 6.4 ug/ml, of standard reference and test vancomycin, were evaluated. The plates were then incubated at 30°C for 18 hours. The diameter of the inhibition zone was measured to the nearest 0.1 mm, using a pair of calipers.

A straight-line graph was subsequently plotted based on the mean diameter of inhibition zones obtained from the standard references, with log dose of antibiotics as the x-axis and the diameter of inhibition zone as the y-axis. The log doses of vancomycin after incubation in various test conditions, were interpolated from the diameter readings obtained, from the standard reference graph. The log dose readings will then be anti-logged (*D_β_*). The formula of calculating the percentage potency was as follows:
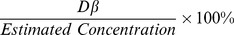



The results obtained were analysed for statistical significance using two-tailed t-test, with the assumption that the population has equal SDs. The results are considered significant if the p-value is less than 0.05.

### Microbiological Results

Small pieces of tissue and 5 ml of solution specimens were randomly sent for routine aerobic, anaerobic and fungal cultures. The methodologies of microbiological and fungal cultures are briefly described in Table 1.

**Table 1:** Methodologies of routine microbiological and fungal cultures.

Type of Culture	Primary Culture	Subculture
**Aerobic**	Specimen was inoculated onto a blood agar plate andMacConkey agar and incubated in 5% carbon dioxide at35°C for 48 hours.	Specimen was incubated in cooked meat broth at 35°C. If the primary cultures had no growth, the cooked meat broth was then subcultured onto blood agar plate and incubated 35°C for 48 hours. If there is no growth, a final subculture is done at the end of 1 week.
**Anaerobic**	Specimen was inoculated onto a CDC anaerobic bloodplate, and incubated in an anaerobic chamber at 35°Cfor 96 hours.	Specimen was incubated in thioglycollate broth in an anaerobic chamber at 35°C. If the primary cultures had no growth, the thioglycollate broth was then subcultured in CDC anaerobic blood plate and incubated at 35°C for 96 hours.
**Fungal**	Specimen was inoculated onto Sabouraud dextrose agar(SDA) and incubated at 30°C for 4 weeks.	Specimen was incubated in brain-heart infusion broth in ambient air at 30°C for 3 days. If the primary culture had no growth, brain-heart infusion broth would be subcultured onto SDA and incubated at 30°C for 4 weeks from time of specimen receipt.

The outcomes of microbiological tests in NCHB’s clinical cardiovascular homografts (which consists of aortic, pulmonary, mitral and tricuspid valves, ascending and descending aorta and pulmonary patches) processed from February 2008 to May 2012, were retrospectively reviewed.

Results of post-recovery and post-antibiotic incubation tissue cultures of homografts processed using the two different antibiotic regimens of penicillin-streptomycin (2008 to 2009) and amikacin-vancomycin (2010 to 2012) were compared. Antibiotic susceptibility results for bacteria isolated in tissue and solution samples during penicillin-streptomycin regimen were also analysed.

## Results

### Antibiotic Potency Assays

#### 1. Antibiotic potencies after 6-hours incubation at 4°C and 37°C

Acceptance criteria for unreduced antibiotic activity was stipulated to be no less than 80% and shall not exceed 125%. Potency results from incubation of vancomycin at 4°C and 37°C for 6 hours were within the acceptable criteria. The difference in potency after incubation in the two temperatures is not considered to be statistically significant (p-value  =  0.32). Hence, we concluded that the potency of vancomycin remained unaffected and its bactericidal activity remained effective after 6-hours incubation at both 4°C and 37°C.

However, the results for amikacin illustrated that while the activity of amikacin remained unaffected at 4°C after 6 hours of incubation, its potency plummeted by 44.9% at 37°C, as compared to the control. This indicated a significant reduction in bactericidal activity at higher temperature (p-value  =  0.015) (Table 2).

**Table 2:** Potencies of antibiotics after subjecting them to different incubation time and temperatures.

Conditions	Vancomycin/Percentage of Potency			Amikacin/Percentage of Potency		
	1^st^ test	2^nd^ test	Average	1^st^ test	2^nd^ test	Average
**0 hour (control)**	102.77	-	-	102.55	-	-
**4^o^C at 6 hours**	99.89	109.27	104.58	107.52	119.09	113.31
**37°C at 6 hours**	100.49	93.57	97.03	61.25	54.01	57.63
**4°C at 24 hours**	105.11	107.17	106.14	75.70	65.55	70.63

Based on this first set of results, it was determined that incubating both antibiotics at 4°C retained their original potencies. Hence, this temperature was used in the second set of tests.

#### 2. Antibiotic potencies after incubation in 4°C for 24 hours

The potency and bactericidal activity of vancomycin were comparable after incubation at 4°C for 6 hours and 24 hours (p-value  =  0.78).

In contrast, incubating amikacin at 4°C for 24 hours had caused a reduction (31.9%) in potency as compared to the control. This signified a decrease in activity of amikacin as incubation duration increases (p-value  =  0.03). However, the extent of degeneration in potency is less than the incubation of amikacin at 37°C for 6 hours (Table 2).

### Review of Microbiological Results

In 2008 and 2009, 36 cardiovascular homografts were decontaminated with penicillin and streptomycin. Among them, 5 homografts (13.9 %) had bacteria isolated post-recovery before antibiotic incubation, but was negative in post-antibiotic incubation culture. A homograft which was tested MRSA positive in the post-recovery tissue was discarded (Table 3).

**Table 3:** Type of micro-organisms isolated from cardiovascular homografts from 2008 to 2012.

Homograft	Post-Recovery Tissue or Solution Culture	Post-Antibiotic Incubation Tissue or Solution Culture
**Penicillin & Streptomycin (February 2008 – December 2009), ** ***n*** ** = 36**		
1	Negative	* *Propionibacterium acnes*
2	*Pseudomonas species*	Negative
3	*Coagulase-negative Staphylococcus,* *Escherichia coli*	Negative
4	*Acinetobacter species*	Negative
5	Negative	* *Micrococcus species*
6	# * *MRSA*	Negative
7	*Rhodococcus species,* *Staphylococcus aureus,* *Propionibacterium acnes*	Negative
8	*Staphylococcus aureus,* *Propionibacterium acnes*	Negative
9	Negative	* *Candida parapsilosis*
**Amikacin & Vancomycin (January 2010 to May 2012), ** ***n*** ** = 35**		
10	*Coagulase-positive Staphylococcus*	Negative
11	*Coagulase-positive Staphylococcus*	Negative
12	*Coagulase-negative Staphylococcus*	Negative
13	*Coagulase-negative Staphylococcus*	Negative
14	*Alpha-haemolytic Streptococcus*	* *Candida albicans*
15	*Proponiobacterium acnes*	Negative
16	*Bacillus species,* *Coagulase-negative Staphylococcus,* *Proponiobacterium acnes*	Negative
17	*Proponiobacterium acnes*	Negative
18	*Proponiobacterium acnes*	Negative
19	* *Aspergillus fumigatus*	Negative
20	* *Candida tropicalis*	Negative
21	*Coagulase-negative Staphylococcus*	Negative

# refers to bacteria, which when isolated at any stages of processing, will have to be discarded.

* refers to cardiovascular homografts that are not for clinical use due to their positive microbiological results.

The results from the antibiotic susceptibility tests of significant pathogenic bacterial isolates revealed that all micro-organisms except for *Micrococcus* species were resistant to penicillin. However, all micro-organisms tested remained susceptible to amoxillin-clavulanic acid and piperacillin-tazobactam, which are penicillins combined with beta-lactam/beta-lactamase inhibitors. Susceptibility testing was conducted using newer aminoglycosides gentamicin and amikacin, while streptomycin was not tested. It was discovered that a strain of *Coagulase-negative Staphylococcus*, a common micro-organism isolated in our tissue and solution samples, was resistant to gentamicin, which is an antibiotic used in some tissue banks to decontaminate tissues. Amikacin susceptibility was only tested for *Acinetobacter species* and both strains were sensitive to it. All the 3 micro-organisms tested for vancomycin susceptibility were sensitive to it (Table 4).

**Table 4:** Antibiotic susceptibility results of bacteria isolated in 2008 and 2009 during penicillin-streptomycin regimen.

Homograft & BacterialIsolate	2	3			4		5	6		7
Antibiotics Tested	PS	CNS Strain 1	CNS Strain 2	EC	AC Strain 1	AC Strain 2	MC	RC	SA	SA
**Penicillin**										
**Penicillin**		R	R				S	R	R	R
**Ampicillin**	R	R	R	R	S	S	S		R	R
**Cloxacillin**		R	S				S		S	S
**Amoxillin- Clavulanic acid**	S			S	S	S				
**Piperacillin- Tazobactam**	S			S	S	S				
**Aminoglycoside**										
**Gentamicin**	S	S	R	S	S	S				
**Amikacin**					S	S				
**Glycopeptide**										
**Vancomycin**		S	S					S		

Abbreviations: PS: *Pseudomonas* species; CNS: *Coagulase-negative Staphylococcus*; EC: *Escherichia coli*; AC: *Acinetobacter* species; MC: *Micrococcus* species; RC: *Rhodococcus* species; SA: *Staphylococcus aureus*; S: Sensitive; R: Resistant.

From January 2010 to May 2012, 35 homografts were decontaminated with new antibiotic combination of amikacin and vancomycin. 11 homografts (33.3 %) were initially tested positive for microbiological culture post-recovery. The higher rate of positive culture post-recovery was attributed to a change in process in 2010 where the heart valve block, consisting of both the aortic and pulmonary valves, was transported in the same solution. This change was implemented to meet the AATB guidelines requiring tissue dissection to be performed in an ISO Class 5 environment. Once a positive post-recovery transport solution culture was reported, it is reflected in both the aortic and pulmonary valves findings. Till date, despite the higher positive culture post-recovery, there has been no case of positive bacterial culture post-antibiotic incubation. However, 3 homografts were tested positive for fungi, mandating their discard (Table 3).

## Discussion

In 2009, NCHB evaluated the effectiveness of penicillin and streptomycin decontamination regimen as several organisms isolated during this period were resistant to one or both these antibiotics. Furthermore, the instability of penicillin in aqueous solution was known since its discovery by Fleming [5]. Results of our penicillin stability study using iodometric assay as stated in USP 31 confirmed our suspicion that our previous antibiotic decontamination regimen might have resulted in significant degeneration in penicillin potency possibly due to the instability of the penicillin solution at 37°C. This may be one of the reasons why the decontamination efficacy of penicillin-streptomycin combination may not be as effective.

Furthermore, there is a high incidence of penicillin resistance among bacteria recovered from our clinical samples. This is part of the well-known worldwide problem of rising antimicrobial resistance. For instance, by the 1950s to 1970s, a consistent prevalence of 65% to 85% of penicillinase-producing *Staphylococcus aureus* strains was discovered in Copenhagen and the United States. Since the introduction of penicillin in 1941, it took 15 to 20 years for the prevalence rates of resistance to reach 25% in the community [6].

In our microbiological surveillance, our team analysed the species of bacterial isolates from tissue and solution samples, as well as their antibiotic susceptibilities from 2008 to 2009. The kind of antibiotics tested is dependent on the type of bacteria recovered according to international guidelines and the availability of antibiotics in our hospital formulary. As such, the various bacteria are tested for susceptibilities to only specific antibiotics.

In general, aminoglycosides are bactericidal and are active in-vitro against a wide spectrum of aerobic and facultative Gram-negative bacilli [7]. The first aminoglycoside discovered in 1943, streptomycin, currently has limited usage in clinical medicine due to widespread drug resistance [8], [9]. The results of this review favoured the substitution of streptomycin with another newer aminoglycoside, amikacin. Due to the presence of the amino-hydroxy-butyryl group, this prevents enzymatic modification of amikacin at multiple positions. This renders amikacin a more effective decontaminating agent as micro-organisms which develop resistance to other aminoglycosides remain susceptible to amikacin [10]. This also gives amikacin a broader spectrum than the other aminoglycosides currently in use. For instance, a study conducted in Egypt revealed that the isolates of Gram-negative bacteria in clinical samples exhibited maximal resistance against streptomycin at 83.4%, minimal resistance against amikacin at 17.7% and intermediate resistance against the other aminoglycosides. It was also discovered that amikacin was more active against Gram-negative bacteria than the other aminoglycosides tested [11].

Apart from the types of antibiotics used, the temperature of incubation also determines the efficacy of decontamination of the homografts. Our results suggested that incubation of tissues with vancomycin-amikacin combination at 4°C for 24 hours provides optimal potency for this decontamination regimen. This was especially true for amikacin, whose potency was observed to be significantly reduced at 37°C in our study. This is an interesting finding, as although amikacin appeared to be heat-labile in our study, to the best of our knowledge, there is no other similar study to support our results. However, a study by Traub et. al. revealed that all aminoglycosides, including amikacin, proved to be heat-stable when the minimum inhibitory concentrations were examined at non-clinical conditions of 56°C and 121°C [12]. This discrepancy could be due to differences in methodologies. Besides, both studies conducted by our team and Traub’s team employed microbiological assays. For all microbiological assays, it is inevitable that the testing of amikacin potency using turbidimetric assay is affected by the growth response of micro-organisms which varies in individual tests. Furthermore, there are some disadvantages of turbidimetric assay, which include more manipulation and greater susceptibility to variations in environmental conditions and extraneous substances in sample preparations, which may influence the growth of tested micro-organisms [13].

It is believed the maximum antibiotic activity is achieved by the incubation of homograft at a warmer physiological temperature of 37°C, when most micro-organisms are actively replicating [3], [14]. This theory was further supported by Germain et. al. that antibiotic incubation at 37°C saw a rapid reduction in the number of colony-forming units of 12 bacterial strains. From the study, all strains with the exception for *Bacillus subtilis,* became undetectable after 12 hours of incubation [3]. In our study, amikacin appeared to be less potent and there might possibly be a compromise to its bactericidal activity at 37°C. Therefore, NCHB selected 4°C as the temperature used during vancomycin-amikacin decontamination. In addition, the advantage of incubating homografts at this temperature is that bacterial proliferation is halted and tissue degradation is reduced, as lysosomal activity is lower at 4°C than at 37°C [15]. A glycopeptide, vancomycin was also included in the new antibiotic regimen because it is effective against most Gram-positive cocci, Gram-positive bacilli [16], including methicillin-resistant strains and Gram-positive anaerobes such as *Propionibacterium* species [17].

Since 2010, the implementation of amikacin and vancomycin as decontaminating agents appeared to reduce the incidence of positive microbiological result in post-antibiotic incubation tissue and solutions cultures for homografts which were initially tested positive in post-recovery cultures. However, it is difficult to attribute an increased effectiveness to a change in antibiotic regimen due to several reasons: (1) The sample sizes were small. (2) There was no conclusive data to demonstrate a failure in decontamination of penicillin-streptomycin regimen. This was because there was no case of positive post-antibiotic incubation tissue or solution culture for a microbe that had been isolated post-recovery, which would have been an evidence of failure. In the cases of *Propionibacterium acnes* and *Micrococcus* species, which had been isolated post-antibiotic incubation, the micro-organisms were not found in post-recovery cultures. This suggested they might have been introduced during processing after antibiotic decontamination. This appeared to be the case for *Micrococcus* species, as the strain was susceptible to the decontaminating agent, penicillin. (3) The method of preparation of post-recovery solution culture was changed at about the same time as the use of new amikacin-vancomycin regimen. This makes it difficult to ascribe the variation in culture results to either of these two changes.

It is imperative to monitor microbiological trends closely and evaluate the efficacy of current antibiotics regimen against emerging resistant strains of micro-organisms. NCHB is aware of the ineffectiveness of current regimen against fungus, and will include an antifungal drug should the trend of fungal recovery changes in the future. Another aspect to note in conjunction with microbiological testing is the sampling technique used in the collection of tissue and solution specimens for culture. An effective method shall give an accurate result by improving the microbial detection rate and eliminating the potential of inhibitory effect caused by residual antibiotics [18]. Due to this potential for inhibition of bacterial and/or fungal growth, antibiotics and sterility test must also be validated to ensure that any bacteriostatic and/or fungistatic properties of the tissues and solutions do not adversely affect the reliability of the test results caused by false-negatives [19].
